# Expert-level aspiration and penetration detection during flexible endoscopic evaluation of swallowing with artificial intelligence-assisted diagnosis

**DOI:** 10.1038/s41598-022-25618-z

**Published:** 2022-12-15

**Authors:** Weihao Weng, Mitsuyoshi Imaizumi, Shigeyuki Murono, Xin Zhu

**Affiliations:** 1 Graduate School of Computer Scicence and Engineering, The University of Aizu, Aizuwakamatsu, 965-8580 Japan; 2 Department of Otolaryngology, Fukushima Medical University, Fukushima, 960-1295 Japan

**Keywords:** Image processing, Biomedical engineering, Translational research, Oral diseases

## Abstract

Flexible endoscopic evaluation of swallowing (FEES) is considered the gold standard in diagnosing oropharyngeal dysphagia. Recent advances in deep learning have led to a resurgence of artificial intelligence-assisted computer-aided diagnosis (AI-assisted CAD) for a variety of applications. AI-assisted CAD would be a remarkable benefit in providing medical services to populations with inadequate access to dysphagia experts, especially in aging societies. This paper presents an AI-assisted CAD named FEES-CAD for aspiration and penetration detection on video recording during FEES. FEES-CAD segments the input FEES video and classifies penetration, aspiration, residue in the vallecula, and residue in the hypopharynx based on the segmented FEES video. We collected and annotated FEES videos from 199 patients to train the network and tested the performance of FEES-CAD using FEES videos from other 40 patients. These patients consecutively underwent FEES between December 2016 and August 2019 at Fukushima Medical University Hospital. FEES videos were deidentified, randomized, and rated by FEES-CAD and laryngologists with over 15 years of experience in performing FEES. FEES-CAD achieved an average Dice similarity coefficient of 98.6$\%$. FEES-CAD achieved expert-level accuracy performance on penetration (92.5$\%$), aspiration (92.5$\%$), residue in the vallecula (100$\%$), and residue in the hypopharynx (87.5$\%$) classification tasks. To the best of our knowledge, FEES-CAD is the first CNN-based system that achieves expert-level performance in detecting aspiration and penetration.

## Introduction

Dysphagia is a disorder of swallowing commonly occurring in patients with strokes and neurological diseases^1^. Oropharyngeal dysphagia is typically caused by abnormalities of muscles, nerves, or structures of the oral cavity, pharynx, or the sphincter at the top of the esophagus, and about 30–50$\%$ of stroke patients may suffer from longstanding oropharyngeal dysphagia^2,3^. Oropharyngeal dysphagia after stroke is the root cause of health complications like aspiration pneumonia, dehydration, and malnutrition. Aspiration and penetration are the most severe complications caused by oropharyngeal dysphagia. *Aspiration* is defined as the passage of material below the vocal fold (into the subglottis), and *penetration* is the passage of material into the laryngeal vestibule but not into the subglottis. Jaderberg et al.^4^ suggest that mild aspiration in healthy adults may not bring about medical complications. However, Crausaz et al.^5^ record that a degree of aspiration in stroke patients can result in serious medical consequences, including bacterial pneumonia, chemical pneumonitis, and even death. As a tertiary prevention after stroke, the prevention and treatment of aspiration are critical in increasing stroke survivors’ survival rates^6^.

Flexible endoscopic evaluation of swallowing (FEES) and videofluoroscopic swallow study (VFSS) represent the gold standards in studying oropharyngeal dysphagia^7^. Considering the lower detection performance of FEES without video recording, FEES with video recording or videofluoroscopy is mainly used to detect aspiration and penetration accurately. Videofluoroscopy can detect aspiration and penetration more accurately but requires radiation exposure. Onofri et al.^8–11^ reported the comparison between the diagnostic values of FEES and VFSS regarding the quality of aspiration and penetration detection. Their experiments show that FEES has many advantages over VFSS: (1) uses test bolus with no radiation exposure or barium; (2) does not limit the exam length; (3) assesses patient endurance during a meal; (4) views the larynx and pharynx directly; and (5) can yield significant performance gain over VFSS in identifying aspiration and penetration. However, accurate detection of aspiration and penetration by FEES after swallowing remains a clinical challenge for inexperienced doctors because white-out and/or motion of swallowing is so fast, usually only lasting 0.3 s^12^. Furthermore, the interpretation results of FEES may vary among examiners with different levels of experience although they are required to evaluate swallowing impairment appropriately. As a result, the interdifference in diagnosis would significantly affect the clinical course of patients with swallowing impairment such as the incidence of aspiration pneumonia after FEES. One potential solution is to detect aspiration and penetration with artificial intelligence (AI)-assisted FEES.

To date, studies of AI-assisted methods have been performed using traditional machine learning methods^13^ and traditional image-based AI-assisted medical equipment^14^. However, traditional supervised learning models like support vector machines have not reached a satisfactory level of performance and were developed slowly in the past few decades. The breakthroughs of AI-assisted medical equipment took place in the last decade^15^ when researchers utilized a convolutional neural network (CNN) to analyze medical images and achieved the practical standard. Ahsan et al.^16^ developed a method based on CNNs to predict and differentiate between COVID-19 and non-COVID-19 patients. Islam et al.^17^ used a pretrained deep learning model’s weight to detect potato diseases from leaf images. Danala et al.^18^ proposed a deep transfer learning-based CAD to classify breast lesions with a relatively large and diverse retrospective dataset. Attention modules^19^ have arguably become a valuable component in deep learning. Attention mechanism are based in a common-sense intuition that people focus to a certain part when processing a large amount of information. CNN-based systems lack long-range pixel-pixel dependencies that are present in an image^20^. Many efforts have been made on exploiting long-range pixel-pixel dependencies for CNN to model global contextual representations in biomedical images, and achieved significantly improved biomedical image segmentation performance^21–23^. Islam et al.^24^ improved the performance of breast lesion classification with attention mechanisms. Rajpurkar et al.^25^ evaluated CNN-based computer-aided diagnosis (CAD) through competing with four radiologists with 4, 7, 25, and 28 years’ experiences in detecting pneumonia from frontal-view chest X-ray images. The CAD achieved expert-level performance on the F1 metric. Moreover, Tschandl et al.^26^ conducted a web-based comparison between their CNN based CAD and 95 dermatologists in diagnosing skin cancer, and the CAD achieved better performance than that of beginners and intermediate dermatologists but slightly lower performance than that of expert dermatologists with over 10 years’ experiences.

A massive number of annotated data are of prime importance to improve CNN’s performance. Many works^27^ were focused on leveraging images from large-scale nonmedical datasets such as ImageNet^28^. For medicine-related implementation, existing methods can only be retrained on small-scale medical datasets^29^. As a result, CNN has to initialize many hidden layers previously trained on large-scale nonmedical image segmentation to ensure fine-tuned models have promising results^30^. Transfer across acquisition protocols or imaging modalities (domain adaptation) has better performance than that of transfer from nonmedical images. Karimi et al.^31^ improved target task segmentation performance with images acquired by different acquisition protocols or imaging modalities. Ghafoorian et al.^32^ reported a CNN, that was transferred from images by different scanners and fine-tuned on two target samples, achieved an average Dice score of 48 $\%$ over a unpretrained model. Zhu et al.^33^ used generative adversarial networks to transfer knowledge to medical images in a target modality from images in another.

However, each aforementioned domain-adapted CNN requires a feasible large-scale labeled medical dataset. This problem has been partially addressed by recent self-supervised and semisupervised learning methods and applications in medical image analysis. Self-supervised and semisupervised learning approaches aim to learn from carefully chosen unlabeled medical images and then transfer knowledge by finetuning CNN on labeled target medical images. Self-supervised methods^34^ pretrain CNN with unlabeled images. Surrogate task-based self-supervised methods transfer knowledge from discriminating between a set of classes on surrogate tasks, e.g., image inpainting^35^, image colorization^36^, relative pixel matching^37^, jigsaw puzzles solving^38^, and rotation prediction^39^. Contrastive learning^40^ is a popular form of surrogate task-based self-supervised learning. It reduces the distance of sampled pairs from the same surrogate class and enlarges the distance of sampled pairs from different classes.

The generalizability of transferred knowledge by surrogate task-based self-supervised learning relies on building a target-related and easy pretrained task^41^. However, collecting a suitable unlabeled dataset is difficult and may be impossible for a specific target task. The segmentation performance of recent AI-assisted CAD is lower in clinical practice than that in development stages because of data heterogeneity and interobserver variability^42^. The reliability of practical clinical applications at a hospital with a specific acquisition protocol and imaging modality may partially be improved through training by their own annotated datasets even though annotation is expensive and time-consuming.

Despite the prevalence of CNN, existing models^43–45^ and training strategies^46,47^ have rarely been used in detecting aspiration and penetration with FEES. Expert-level automated detection of aspiration and penetration from FEES would be clinically significant for beginner and intermediate clinicians because outward signs or symptoms of dysphagia have inter- and intraindividual differences. Moreover, AI-assisted CAD would be a remarkable benefit in providing healthcare to populations with inadequate access to dysphagia experts, especially in aging societies^48^. This paper presents an AI-assisted CAD that can automatically detect aspiration and penetration by segmenting the vocal fold, subglottis, laryngeal vestibule, test bolus, and white-out on video recording during FEES at an expert-level performance. For the experiment, the proposed FEES-CAD was trained using 25,630 expert-annotated images from 199 complete videos recorded during FEES and tested on 40 FEES videos. To the best of our knowledge, FEES-CAD is the first CNN-based system that achieves expert-level performance in detecting aspiration and penetration^49,50^.

## Background

The proposed AI-assisted CAD named *FEES-CAD* addresses the need for aspiration and penetration detection on FEES by monitoring the movement of test bolus in oropharyngeal regions. Its main task is to segment objects of interest in FEES video frames, i.e., the vocal fold, subglottis, laryngeal vestibule, test bolus, and white-out. This section reviews recent approaches to medical image segmentation and their potential limitations.

**Figure 1:**
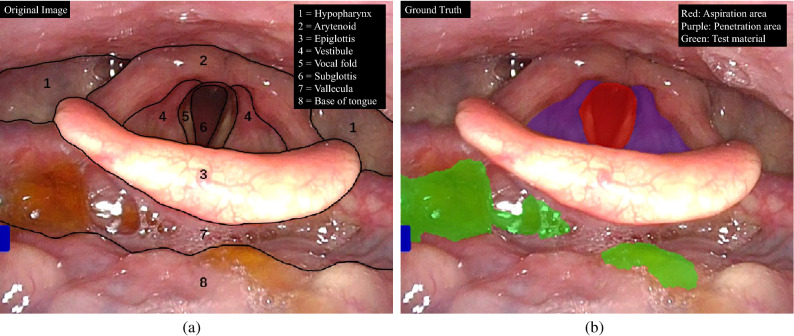
(**a**) Example of the original video frames. Outline of the eight different anatomic landmark structures in a FEES video: the hypopharynx (1), arytenoid (2), epiglottis (3), vestibule (4), vocal fold (5), subglottis (6), vallecula (7), and the base of the tongue (8). (**b**) Example of the segmentation video frames. Annotation of aspiration area (red) created by the two anatomic landmark structures of the vocal fold and subglottis, penetration area (purple) created by the laryngeal vestibule, and annotation of jelly as test bolus (green).

Many techniques have been proposed to realize more accurate segmentation, e.g., attention mechanism^51^, domain knowledge^52^, and uncertainty estimation^53^. In the past decade, CNN plus attention mechanism has been introduced in medical image segmentation. An attention mechanism that simulates the human brain’s capability to sort out information was first introduced by Bahdanau *et al.*^54^ for machine translation. An expansive path was designed to dynamically select useful source language words or subsequences for translation into a target language. Recently, its variant, self-attention^55^, is widely implemented in medical image segmentation. However, self-attention has a fundamental issue in supervised medical image segmentation, where extra parameters have to be introduced to CNN. This makes CNN easy to overfit with the training data. However, medical image segmentation tasks usually require higher accuracy than natural image segmentation tasks. Figure 1a shows an example of the original video frames, and Fig. 1b shows an example of the segmented video frames. The objects of interest include the aspiration area (red) created by the two anatomic landmark structures of the vocal fold and subglottis, the penetration area (purple) created by the laryngeal vestibule, and the annotation of jelly as test bolus (green). Changing the angle and lighting in a video recording during FEES makes some strong outliers in the training data in the test bolus. Classification of test bolus would locate more in the domain of the other classes, and then overfitting causes shifted boundaries of these outliers in some directions. Such shifted boundaries result in high false positives as partial domains belonging to other classes are occupied by the test bolus. Aspiration and penetration detection aims to analyze if the test bolus enters the larynx or even below the true vocal. While a high false positive may be uncritical in some medical tasks, it may cause unreliable aspiration and penetration detection results.

## Methods

### Flexible endoscopic evaluation of swallowing

**Figure 2:**
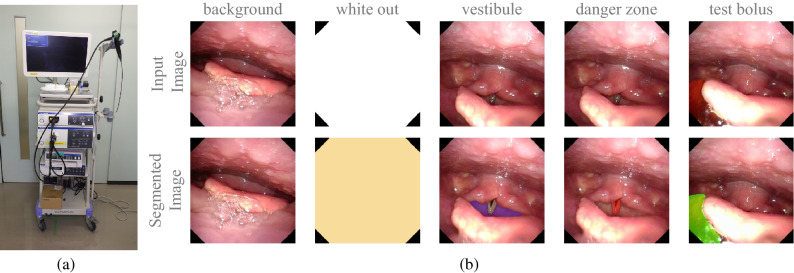
FEES was performed using (**a**) a laryngeal flexible endoscope with a diameter of 2.6 mm (ENF-V3, O, OLYMPUS Corp., Tokyo, Japan). (**b**) From left to right: image only with background,“white-out”, penetration area, aspiration area, and test bolus.

**Figure 3:**
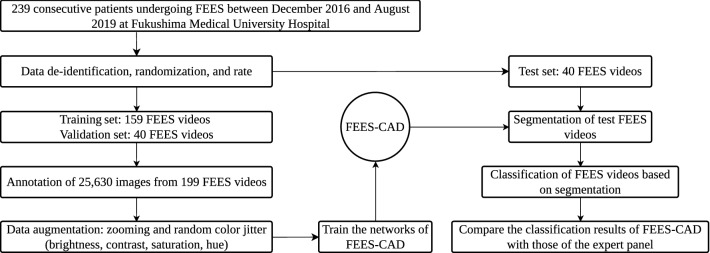
Flowchart of this study.

As shown in Fig. 2a, FEES was performed using a laryngeal flexible endoscope with a diameter of 2.6 mm (ENF-V3, O, OLYMPUS, Corporation Tokyo, Japan), and the corresponding videos were recorded. Figure 3 illustrates the flowchart of this study. The study subjects were consecutive patients with suspected oropharyngeal dysphagia who underwent FEES between December 2016 and August 2019 at Fukushima Medical University Hospital^56^. The research protocol was prepared in accordance with the Declaration of Helsinki, and this research was approved by the Institutional Review Board and Ethics Committee of Fukushima Medical University (2019-154). Each participant signed informed consent for FEES before this examination was conducted. FEES, which was video-recorded, was performed by transnasally passing a laryngeal flexible endoscope. Aspiration and penetration are the most severe complications caused by oropharyngeal dysphagia, of which aspiration is when the passage of food below the vocal fold (into the subglottis), and penetration means the passage of food into the laryngeal vestibule but not into the subglottis. However, it is relatively hard to detect subglottis regions in swallowing endoscopy compared with the vocal fold. In this experiment, the aspiration area is composed of two anatomic landmark structures of the vocal fold and subglottis as it indicates whether food is too close to the windpipe (trachea). As shown in Fig. 1, the original video recording during FEES allows clinicians to investigate the condition of the hypopharynx, arytenoid, epiglottis, vestibule, vocal fold, subglottis, vallecula, and base of the tongue. By contrast, FEES video segmentation highlights the outline of the aspiration area (red), the penetration area (purple), and food,i.e.,test bolus (green). In addition to the mentioned classes, FEES-CAD also recognizes the“none”and “white-out”images Fig.  2b).“White-out”is the overexposed image caused by the reducing distance between the posterior wall of the pharynx and the light from the endoscope, indicating the motion of swallowing. The motion of swallowing is very fast and takes about 0.3 s. Therefore,“white-out”detection is a critical FEES-specific swallowing task. When FEES-CAD segments the “white-out”, it records the starting point for“white-out”and reminds the laryngologists. Therefore, FEES-CAD is designed for a five-class segmentation problem.

**Figure 4:**
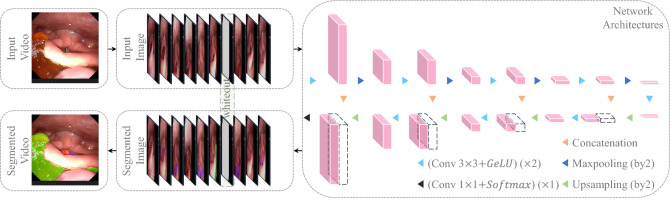
Workflow of the FEES-CAD.

**Table 1:** Characteristics of patient.

	Number	Oral intake	Dysphagia	Age		Height		Weight	
				Mean	Median	Mean	Median	Mean	Median
Training data	159	85	146	63.74	71.00	154.96	162.30	49.84	48.80
Validation set	40	20	34	59.08	69.00	152.65	157.10	46.44	47.20
Test set	40	18	37	64.28	72.50	152.44	160.00	47.13	47.40

*P* value*		Oral intake	Dysphagia	Age		Height		Weight	
Training and validation		0.894	0.589	0.308		0.903		0.931	
Training and test		0.397	0.645	0.842		0.736		0.460	
Validation and test		0.576	0.433	0.515		0.728		0.507	

*$\chi ^{2}$ test was used for oral intake and dysphagia, and *t* test for other characteristics.

**Figure 5:**
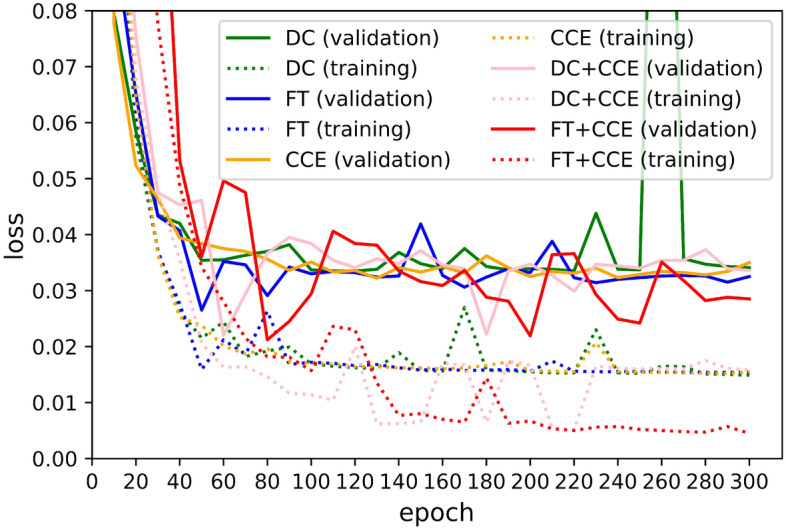
Progress of the training (dotted line) and validation loss (dotted line) with the number of epochs during training with DC (Green), FT (Blue), CCE (orange), DC plus CCE (pink), and FT plus CCE (red) loss functions, respectively.

### Artificial intelligence-assisted computer-aided diagnosis

Figure 4 shows the workflow of the proposed FEES-CAD. FEES-CAD captures video frames from a FEES video and then inputs video frames into a CNN. Implemented CNN is extended from the commonly used architecture named UNet. The network architecture of customized UNet is depicted in Fig. 4(right). It consists of four contracting paths, four expansive paths, and a bottleneck connecting the last contracting path and the first expansive path. Every contracting path, expansive path, and bottleneck has two $3\times 3$ convolutions with strides $1\times 1$, both of which are activated by a Gaussian error linear unit. Each contracting path follows a $2\times 2$ max-pooling with stride $2\times 2$ to extract high-level semantic representations for image recognition. Each contracting path starts with an unpooling with stride $2\times 2$, and the upsampled feature maps concatenate the feature maps derived from the corresponding contracting path. In the contracting path, expansive path, and bottleneck, every convolution follows a batch normalization^57^ before activation. At the end of the customized UNet, a softmax activated $1\times 1$ convolution with strides $1\times 1$ predicts the segmentation outcome. Finally, segmented images convert to a segmented video. The number of channels in the customized UNet architecture starts with 32, doubles after the max-pooling layers, and halves after the unpooling layers.

### Experiments

The network of FEES-CAD was developed using Python 3.9 and the Tensorflow 2.6.0 framework. It was trained on a workstation with an Intel core i7-10700F processor CPU, 32.0 GB RAM, and NVIDIA GeForce RTX 3090.

#### Datasets

The network of FEES-CAD was developed on 25,630 expert-annotated images from 199 FEES videos and tested on 40 FEES videos shown in Table 1. The training and validation set includes 159 and 40 videos randomly selected from the 199 FEES videos for model development. The frame per second (fps) and size of videos recordings are 60 fps and 1920–1080, respectively. The average duration of training and validation videos recordings is 381.1 s, and that of the test videos is 384.8 s. There was no significant difference in diseases, age, height, and weight among the training, validation and test datasets. Videos were deidentified, randomized, and rated by an expert panel of laryngologists as well as dysphagia experts with over 15 years of experience performing FEES. The diagnosis of the underlying disease (primary disease) was confirmed before the test by the expert panel and CAD. The ground truth was determined through repeated video observation and discussion between 2016 and 2019 by laryngologists and dysphagia experts with 20 years’experience in performing FEES and also certificated as swallowing consultation doctors by The Society of Swallowing and Dysphagia of Japan. The expert panel was blinded to all identifying information about examinees and examined the test videos in real time combined with frame-by-frame analysis.

#### Training methodology

The input image size was resized as $256\times 256$. Data augmentations were applied to training imaging through zooming and color jitter to randomly change the value of brightness, contrast, saturation, and hue. The network of FEES-CAD is trained from scratch and initialized with weights by a He-normal initializer^58^. All networks in these experiments use nondifferentiable activation. Hence, it is better to choose the He-normal initializer that provides a more efficient gradient descent than that using a nondifferentiable activation function.

Loss function directly affects training convergence and generalization performance of the network. Most AI-assisted CAD in medical image segmentation was designed for binary segmentation tasks. However, aspiration and penetration detection is a multiclass segmentation problem. FEES video segmentation is also an imbalance problem. To choose a suitable loss function, we performed comprehensive experiments on our dataset using various loss functions. Let $y_{ci}$ and $p_{ci}$ correspond to the ground truth and the output prediction, where *c* and *i* denote class and pixel, respectively. Generalized Dice loss (GDL) ($\mathscr {L}_{\mathrm {GD}}$)^59^ originates from an overlap index named Dice similarity coefficient. GDL has the class re-balancing properties of the generalized Dice overlap and thus achieved good performance on different multiclass segmentation tasks. It can be formally written as

1 $$\begin{aligned} \mathscr {L}_{\mathrm {GD}}=\sum _{c=1}^{C} (1-\frac{w_{c} \sum _{i=1}^{N} y_{c i} p_{c i}}{w_{c} \sum _{i=1}^{N} y_{c i}+p_{c i}}) \end{aligned}$$

where $w_{c}$ provides invariance to different class properties. However, the training stability when using GDL is dependent on the inherent uncertainty in a task^60^. Generalized Tversky loss (GTL)($\mathscr {L}_{\mathrm {GT}}$) addresses this problem by adjusting weights of false positive ($\alpha$) and false negative ($\beta$). GTL is defined as

2 $$\begin{aligned} \mathscr {L}_{\mathrm {GT}}= \sum _{c=1}^{C} (1- \frac{\sum _{i=1}^{N} y_{c i} p_{c i}}{\sum _{i=1}^{N} y_{c i} p_{c i}+\alpha \sum _{i=1}^{N} y_{\bar{c} i} p_{c i}+\beta \sum _{i=1}^{N} y_{c i} p_{\bar{c} i}}) \end{aligned}$$

where $p_{c i}$ denotes the probability of pixel *i* belonging to the class *c* and $p_{\bar{c} i}$ denotes the probability of pixel belonging to other classes. $y_{\bar{c} i}$ is 1 for class *c* and 0 for other classes, and conversely, $y_{c i}$ takes values of 1 for other classes and 0 for class *c*. $\mathscr {L}_{\mathrm {GT}}$ balances the weights for training. By adjusting the hyperparameters $\alpha$ and $\beta$, $\mathscr {L}_{\mathrm {GT}}$ balances the cost of false positives and false negatives. $\mathscr {L}_{\mathrm {GT}}$ is equal to $\mathscr {L}_{\mathrm {GD}}$ in the case of $\alpha = \beta = 0.5$.

Another popular loss function for training multiclass segmentation networks is categorical cross-entropy loss ($\mathscr {L}_{\mathrm {CCE}}$). It computes how distinguishable two discrete probability distributions are from each other, and its gradient is nicer than $\mathscr {L}_{\mathrm {GD}}$ and $\mathscr {L}_{\mathrm {GT}}$. $\mathscr {L}_{\mathrm {CCE}}$ is computed as

3 $$\begin{aligned} \mathscr {L}_{\mathrm {CCE}}(y, p)=-\frac{1}{N} \sum _{c=1}^{C} \sum _{i=1}^{N} y_{ci} \cdot \log \left( p_{ci}\right) . \end{aligned}$$

The loss function of the customized UNet is a combination of $\mathscr {L}_{\mathrm {GT}}$ and $\mathscr {L}_{\mathrm {CCE}}$ to allow for some diversity in the loss while benefiting from the stability of CCE^61^. $\mathscr {L}_{\mathrm {CCE+GT}}$ denotes a weighted sum of $\mathscr {L}_{\mathrm {GT}}$ and $\mathscr {L}_{\mathrm {CCE}}$:

4 $$\begin{aligned} \mathscr {L}_{\mathrm {CCE+GT}}=\gamma \mathscr {L}_{\mathrm {CCE}}-(1-\gamma )\mathscr {L}_{\mathrm {GT}} \end{aligned}$$

The comparison of the loss function is presented in the Supplementary Material. The network of FEES-CAD minimizes loss with an adaptive moment estimation (ADAM) optimizer^62^. ADAM is derived from two stochastic gradient descent approaches, adaptive gradients^63^ and root mean square propagation^64^. It adaptively computes different learning rates for individual parameters from estimates of the first and second moments of the gradients. ADAM optimizer is often the most efficient stochastic optimization in deep learning applications. Figure 5 illustrates the progress of the training (dotted line) and validation loss (dotted line) with the number of epochs during training with DC (Green), FT (Blue), CCE (orange), DC plus CCE (pink), and FT plus CCE (red) loss function. All networks were trained for 100 epochs using an ADAM optimizer with a batch size of 8 because neither of the networks improved after 100 epochs. The initial learning rate is dependent on the networks and decays 1e−4 every five epochs when validation loss is not improved. For a given class *C*, $true \ positive$ is pixel classified correctly as *C*, $false \ positive$ is pixel classified incorrectly as *C*, $true \ negative$ is pixel classified correctly as not *C*, and $false \ negative$ is pixel classified incorrectly as not *C*. The quantitative performance is evaluated by *Accuracy* ($\%$), *Sensitivity* ($\%$), *Specificity* ($\%$), $Dice \ similarity \ coefficient$ (*DSC*, $\%$), and 95$\%$ $Hausdorff \ distance (HD95)$:

5 $$\begin{aligned}&Accuracy= \frac{true \ positive + true \ negative}{(true \ positive+false\ negative) + (true \ negative+false\ positive)} \end{aligned}$$

6 $$\begin{aligned}&Sensitivity= \frac{true \ positive}{(true \ positive+false\ negative)} \end{aligned}$$

7 $$\begin{aligned}&Specificity= \frac{true \ negative}{(true \ negative+false\ positive)} \end{aligned}$$

8 $$\begin{aligned}&DSC=\frac{2 \times true \ positive}{(true \ positive+false\ positive)+(true \ positive + false\ negative)} \end{aligned}$$

9 $$\begin{aligned}&HD95=\max \left\{ \max _{p_{c i} \in P} \min _{y_{c i} \in Y} \ distance(p_{c i}, y_{c i}), \max _{y_{c i} \in Y} \min _{p_{c i} \in P} \ distance(p_{c i}, y_{c i})\right\} . \end{aligned}$$

Aspiration and penetration are historically the only indicators of interest in FEES because they are associated with potentially severe medical consequences^65^. Residue recently has been used as another clinical sign of swallowing dysfunction. Residue in the vallecula and the hypopharynx increases the risk of aspiration and can be aspirated after finishing FEES. Residue stuck in throats can also cause serious symptoms to people with dysphagia^66^ such as weight loss and malnutrition^67^. Our experiments use four indicators for assessing swallowing impairment severity, *penetration, aspiration, residue in the vallecula, and residue in the hypopharynx.* Test dataset consists of 40 videos, and thus the number of all classifications is 160. The expert panel blindly analyzed the original FEES videos of the test dataset to judge the presence or absence of penetration, aspiration, residue in the vallecula, and residue in the hypopharynx, while FEES-CAD classified the segmented FEES videos. The corresponding quantitative classification performance is also evaluated by *accuracy*(%), *sensitivity*(%), and *specificity*(%).

### Ethical approval

The research protocol was prepared in accordance with the Declaration of Helsinki, and this research was approved by the Institutional Review Board and Ethics Committee of Fukushima Medical University (2019-154). Each participant signed informed consent for flexible endoscopic evaluation of swallowing before this examination was conducted.

## Results

FEES-CAD is designed for analyzing FEES videos after patients have undergone FEES. It can be noted that aspiration and penetration detection is not time-bound. FEES-CAD processes images at 3.76 frames per second. The segmentation performance of the customized UNet is shown in Table  2, and the comparison against other networks is presented in the Supplementary Material. Tables  3, 4 and 5 show the comparison of quantitative classification performance between the FEES-CAD and expert panel.

### Results of test FEES videos

**Table 2:** Segmentation results of the customized UNet.

Network	DC loss	FT loss	CCE loss	DSC (%)	Sensitivity (%)	Specificity (%)	HD95	AUC
Customized UNet	$\checkmark$			95.97	96.14	98.95	0.00	0.9787
Customized UNet		$\checkmark$		97.56	97.84	99.32	0.00	0.9880
Customized UNet			$\checkmark$	97.25	97.82	99.16	0.00	0.9871
Customized UNet	$\checkmark$		$\checkmark$	98.07	98.38	99.43	0.00	0.9908
Customized UNet		$\checkmark$	$\checkmark$	98.63	98.56	99.68	0.00	0.9925

As shown in Table  2, customized UNet manages semantic segmentation with a high level of performance. For test FEES videos, we observed that the FT and CCE loss are optimum for obtaining accurate pixel-level segmentation. When the customized UNet was trained on a combination of FT and CCE loss, it achieved the highest segmentation performance based on DSC (98.63$\%$), sensitivity (98.56$\%$), specificity (99.68$\%$), and HD95 (0.00). This high-quality segmentation guarantees satisfactory classification performance.

**Table 3:** Comparison between FEES-CAD and expert panel on the overall classification.

	Overall		
	Accuracy (%)	Sensitivity (%)	Specificity (%)
Expert panel	97.50	90.91	100.00
FEES-CAD	93.13	95.45	92.24

**Table 4:** Comparison between FEES-CAD and expert panel on penetration and aspiration classification.

	Penetration			Aspiration		
	Accuracy (%)	Sensitivity (%)	Specificity (%)	Accuracy (%)	Sensitivity (%)	Specificity
Expert panel	97.50	50.00	100.00	97.50	66.67	100.00
FEES-CAD	92.50	100.00	92.11	92.50	66.67	94.59

**Table 5:** Comparison between FEES-CAD and expert panel on residue in the vallecula and residue in the hypopharynx classification.

	Residue in the vallecula			Residue in the hypopharynx		
	Accuracy (%)	Sensitivity (%)	Specificity (%)	Accuracy (%)	Sensitivity (%)	Specificity (%)
Expert panel	95.00	88.24	100.00	100.00	100.00	100.00
FEES-CAD	100.00	100.00	100.00	87.50	93.75	83.33

**Figure 6:**
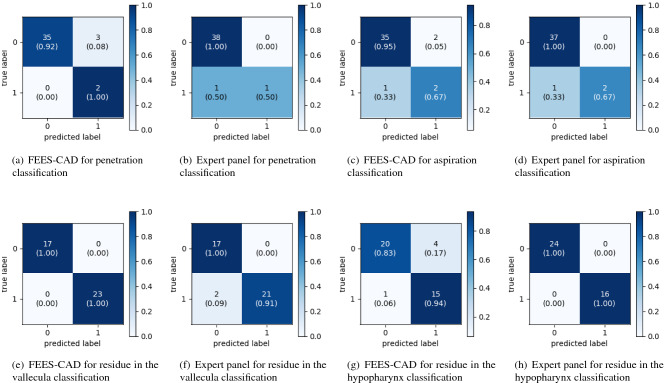
Confusion matrices for visualizing classification performance.

Table 3 lists the classification results of the expert panel and FEES-CAD. We have three major observations from Table 3. First, FEES-CAD achieved an average of 95.45$\%$ sensitivity for overall classification (penetration, aspiration, residue in the vallecula, and residue in the hypopharynx), outperforming the expert panel. FEES-CAD provides outlines of objects of interest as it makes the detection and following of test bolus easier and is very useful in aspiration and penetration detection. Second, FEES-CAD reached a satisfactory level of performance in accuracy and specificity. Particularly, the expert panel outperformed FEES-CAD in specificity because dysphagia experts carefully examined the FEES videos multiple times to prevent false positives. Finally, FEES-CAD is more sensitive than the expert panel. Therefore, FEES-CAD can provide complementary information to improve the aspiration and penetration detection performance of clinicians.

Tables 4 and 5 show the evaluation of FEES-CAD for each classification task. Penetration and aspiration classifications are the main tasks. A patient is classified into penetration or aspiration only when the examiner can find a test bolus entering the laryngeal vestibule or the subglottis in the FEES video. FEES-CAD achieved 100$\%$ on penetration classification, which is significantly better than that of the expert panel. In addition, FEES-CAD reached the same level of performance on aspiration classification. Residue in the vallecula and residue in the hypopharynx classification are also important because the residue may enter the vestibule and subglottis after finishing FEES and thus increases the risk of penetration and aspiration. FEES-CAD can perfectly classify the residue in the vallecula. Among all tasks, residue in the hypopharynx classification is the only one for which the expert panel attains higher sensitivity.

**Figure 7:**
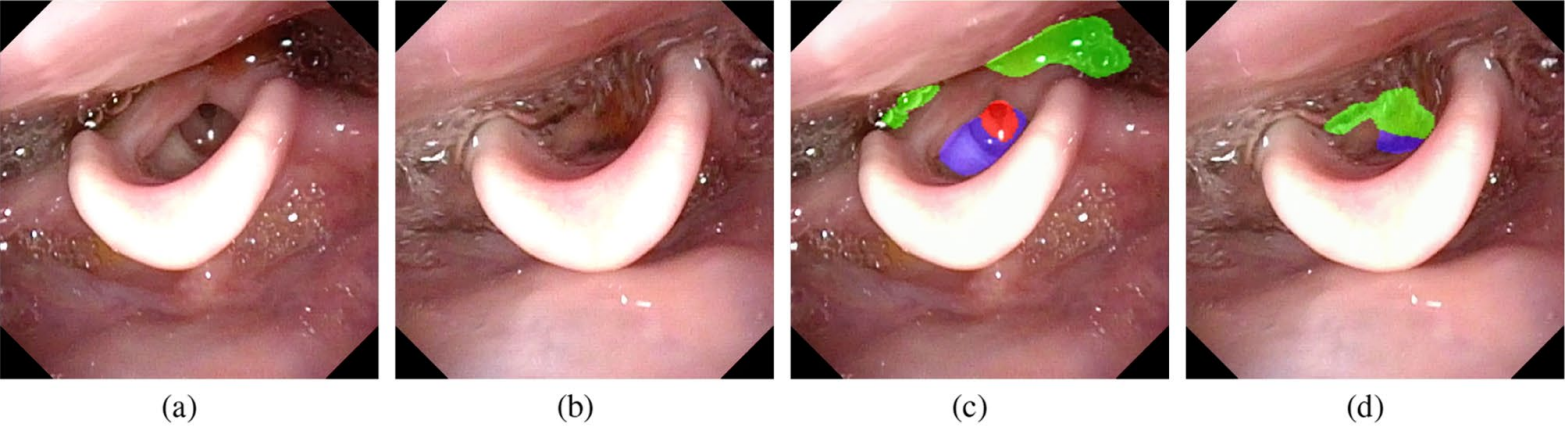
An example of (**a, b**) two consecutive FEES images and (**c, d**) their corresponding segmentation results.

## Discussion

**Figure 8:**
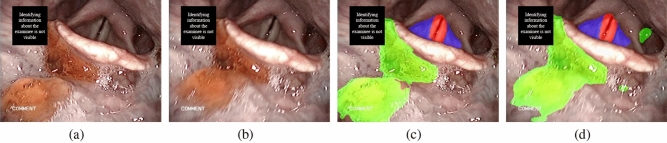
An example of two consecutive FEES images (**a**) and (**b**), and their corresponding segmentation results (**c**) and (**d**). Red: aspiration area, Purple: penetration area, and Green: test bolus.

The shortage of dysphagia experts and raters to rate FEES for diagnostic purposes has become a serious social problem^68,69^. The lack of timely diagnosis and treatment of dysphagia results in longer hospital lengths of stay, longer rehabilitation time, higher mortality, and higher healthcare expense^69–71^. Figure 6 illustrates confusion matrices of classification by expert panel and FEES-CAD. From the comparison between FEES-CAD and the expert panel shown in Tables 3–5, we found that the FEES-CAD achieves satisfactory results and outperforms the expert panel on several tasks. Because FEES-CAD guarantees a high sensitivity with reasonable specificity, it can be adopted in clinical practice to provide a timely and accurate diagnosis.

FEES-CAD demonstrated higher sensitivities and outperformed the expert panel on several tasks. The major problem for the expert panel in improving the classification performance is that they sometimes lose tracking. Figure 7 shows an example of two consecutive FEES images in Fig. 7a,b and their corresponding segmentation results in Fig. 7c,d. These images were extracted from a test video where FEES-CAD outperformed the expert panel. In this example, a small amount of the test bolus is stuck in the hypopharynx and covered by saliva. The expert panel could not detect penetration and aspiration in this case. Because the motion of swallowing was so fast and only took about 0.3 s, the expert panel hardly performed reliable test bolus trajectory tracking. The expert panel sometimes showed lower performance in reliable test bolus trajectory tracking compared with AI. FEES-CAD alleviates the lost tracking problem by using various colors to make objects stand out and therefore enhance the boundaries of interest. FEES-CAD is proven to have great potential in penetration and aspiration detection. More specifically, FEES-CAD achieves 100.00$\%$ sensitivity, which is significantly better than that of the expert panel, on penetration detection.

FEES-CAD demonstrated lower performance in the detection of residue in the hypopharynx compared with the expert panel. As shown in Fig. 8, we use two consecutive FEES images in Fig. 7a,b and their corresponding segmentation results in Fig. 7c,d to analyze how the expert panel outperformed FEES-CAD. There were residues in the vallecula and the hypopharynx in these consecutive FEES images. Contrast mediums were added to test bolus during FEES to increase the contrast of fluids in the video recording during FEES and thus enhance the detection of residue and airway invasion (aspiration and penetration). However, residue in the hypopharynx might be covered by saliva, and the hypopharynx may be not properly illuminated because of changing angle and lightning from the FEES. UNet learns features pixel-wise and is sensitive to color contrast when distinguishing different objects because UNet can not maintain global spatial and multilevel semantic representations. Thus, UNet has difficulty classifying the liquid in the hypopharynx in some frames. When the network of FEES-CAD wrongly segments some pixels in the hypopharynx as residue, the FEES-CAD is misled to wrongly classify the case as residue in the hypopharynx. In FEES videos, the expert panel could avoid such mistakes based on clinical experience and demonstrates 0$\%$ false positives in all classification tasks. Therefore, the expert panel outperforms FEES-CAD by a large margin on the residue in the hypopharynx classification.

In contrast to residue in the hypopharynx, residue in the vallecula is more likely to be properly illuminated. Therefore, the network of FEES-CAD can segment the residue in the vallecula with a high color contrast. The expert panel cannot recognize all residue in the vallecula caused by the fast motion of swallowing. Therefore, FEES-CAD achieves higher sensitivity and accuracy than the expert panel on the residue in the hypopharynx classification. Similarly, FEES-CAD achieves better performance on penetration because the vestibule is properly illuminated while the aspiration area, especially the subglottis, is often not properly illuminated. FEES-CAD is proven to have great potential in penetration and residue in the hypopharynx classification.

### Limitations and future works

The proposed networks can distinguish test bolus, i.e., the most frequently used jelly-like test food in FEES^72^. The segmentation of other test boluses, such as colorful water, may improve the generalization and clinical usefulness of the proposed method. We still have to tackle the problem of oral intake of saliva, which may influence the observation of test bolus. The customized UNet is the most suitable network for our FEES video segmentation task. However, the segmentation performance of the customized UNet decreases when it is trained with more liquid classes. We can modify the customized UNet to enhance the segmentation performance in more liquid classes.

The proposed FEES-CAD should be improved to automatically classify aspiration and penetration. During the swallow, the pharynx and larynx are invisible when a white-out takes place. White-out is related to the decreasing distance between the pharyngeal walls and the light from the endoscope (pharyngeal constrictor muscle contraction)^73^. Therefore, in the two-dimensional FEES video, the test bolus can generally be observed easily if it appears in the hypopharynx and vallecula but cannot be detected if it enters the airway. In addition, in the proposed network it is difficult to judge whether the test bolus is stuck in the aspiration and penetration areas only using two-dimensional FEES videos. To overcome this problem, three-dimensional reconstruction from two-dimensional images may ease the detection procedure^74^.

The proposed FEES-CAD may be further improved in detecting residues in the hypopharynx. The structures of laryngeal and pharyngeal reduce endoscopy performance when the hypopharynx, vallecula, aspiration areas, and penetration areas are simultaneously illuminated. However, without proper illumination, it is also difficult to label residues in the hypopharynx to train the network. Therefore, we should perform the FEES with proper illumination on the hypopharynx when the test bolus is mixed with other liquids.

## Conclusion

In this paper, we proposed FEES-CAD, a deep learning-based CAD, to analyze the FEES video for the detection of penetration, aspiration, residue in the vallecula, and residue in the hypopharynx. We collected and annotated the FEES videos from patients who underwent FEES between December 2016 and August 2019 at Fukushima Medical University Hospital to train and test the performance of FEES-CAD. Comprehensive experiments on multiple classification tasks show that FEES-CAD is effective in analyzing the FEES video. We also discussed the influence of the changing angle and lightning from the FEES on the performance of the FEES-CAD. FEES-CAD analyzes images pixel-wise and is sensitive to color contrast when distinguishing objects. Therefore, FEES-CAD yields better results when FEES videos are recorded in proper illumination.

## Supplementary Information

Supplementary Information 1.

Supplementary Video S1.

Supplementary Legends.

Supplementary Legends.

Supplementary Video S2.

## Data Availability

The datasets generated and/or analyzed during the current study are not publicly available due to the restrictions of Institutional Review Board but are available from a corresponding author (Mitsuyoshi Imaizumi, ima-mitu@fmu.ac.jp) on reasonable request.
