# Supplementary material for: Expert-level aspiration and penetration detection during flexible endoscopic evaluation of swallowing with artificial intelligence-assisted diagnosis
Source: Sci Rep. 2022 Dec 15;12:21689. doi: 10.1038/s41598-022-25618-z (PMC9753025; doi:10.1038/s41598-022-25618-z)
Supplement: Supplementary file 1 — Supplementary Information 1. [file 41598_2022_25618_MOESM1_ESM.pdf]

Supplementary Material for Expert-Level Aspiration and Penetration Detection During Flexible Endoscopic Evaluation of Swallowing with Artificial Intelligence-Assisted Diagnosis

Weihao Weng[1,+], Mitsuyoshi Imaizumi[2,*,+], Shigeyuki Murono[2], and Xin Zhu[1,*]

[1]Graduate School of Computer Scicence and Engineering, The University of Aizu, Aizuwakamatsu, 965-8580, Japan
[2]Department of Otolaryngology, Fukushima Medical University, Fukushima,960-1295,Japan
[*]ima-mitu@fmu.ac.jp,zhuxin@u-aizu.ac.jp
[+]these authors contributed equally to this work

ABSTRACT

In this supplementary material, we report the qualitative and quantitative comparison of the customized UNet with all the competing networks on the test data for the segmentation of images from flexible endoscopic evaluation of swallowing videos. We also provide comprehensive empirical evidence showing that combining generalized Tversky loss and categorical cross-entropy loss can improve the segmentation performance in this multi-class segmentation task.

Experiments

A series of breakthroughs in image classification using CNN have laid a foundation for networks for semantic segmentation, and the continuous optimization of networks for semantic segmentation has gradually improved the accuracy of medical image segmentation. For baseline comparisons, we first run experiments on both convolutional and transformer-based methods. For convolutional baselines, we used HRNet[1], DeepLabv3+[2], VNet[3], UNet++[4], ResUNet-a[5], and U^{2}Net[6]. For transformer-based baselines, we used three state-of-the-art methods named Att UNet[7], TransUNet[8], and Swin-UNet[9]. All networks was developed using Python 3.9 and the Tensorflow 2.6.0 framework, and was trained on an Intel Core i7-10700F processor CPU, 32.0 GB RAM, and GeForce RTX 3090.

Datasets

All networks was trained by 25,630 expert-annotated images from 199 FEES videos and tested on 40 FEES videos. Videos were deidentified, randomized, and rated by an expert panel of laryngologists as well as dysphagia experts with over 15 years of experience performing FEES. The expert panel was blinded to all identification information about the examinees and examined the videos in real time combined with frame-by-frame analysis.

Training Methodology

The images size are set as 256×256, and training images were augmented through zooming and random color jitter (brightness, contrast, saturation, and hue). All networks were trained from scratch and initialized weights with the He-normal initializer[10].

Let y_{ci} denote a one-hot encoding scheme of ground truth, where p_{ci} is the predicted value by the prediction model for each class, and indices c and i iterate over all classes and pixels, respectively. Generalized Dice loss ($\mathscr{L}_{\text{GD}}$)[11], generalized Tversky loss ($\mathscr{L}_{\text{GT}}$), and categorical cross-entropy loss ($\mathscr{L}_{\text{CCE}}$) are computed as follows,

$$\mathscr{L}_{\text{GD}} = \sum_{c=1}^{C} (1 - \frac{w_c \sum_{i=1}^{N} y_{ci} p_{ci}}{w_c \sum_{i=1}^{N} y_{ci} + p_{ci}}) \tag{1}$$

$$\mathscr{L}_{\text{GT}} = \sum_{c=1}^{C} (1 - \frac{\sum_{i=1}^{N} y_{ci} p_{ci}}{\sum_{i=1}^{N} y_{ci} p_{ci} + \alpha \sum_{i=1}^{N} y_{\bar{c}i} p_{ci} + \beta \sum_{i=1}^{N} y_{ci} p_{\bar{c}i}}) \tag{2}$$

Table S1. Performance Comparison.

Type	Network	DC loss	FT loss	CCE loss	DSC	Sensitivity	Specificity	HD95
Convolutional Baselines	HRNet	✓			94.82	95.58	98.50	1.00
	HRNet		✓		95.85	95.95	98.93	0.00
	HRNet			✓	95.77	95.72	98.96	0.00
	HRNet	✓		✓	95.47	95.47	98.87	0.00
	HRNet		✓	✓	95.59	95.59	98.98	0.00
	DeepLabv3+	✓			93.82	93.82	98.46	1.00
	DeepLabv3+		✓		94.90	96.88	98.18	1.00
	DeepLabv3+			✓	98.20	98.76	99.41	0.00
	DeepLabv3+	✓		✓	97.80	97.99	99.40	0.00
	DeepLabv3+		✓	✓	98.53	**99.58**	98.71	0.00
	VNet	✓			95.66	95.85	98.86	1.00
	VNet		✓		95.67	98.87	95.86	1.00
	VNet			✓	95.62	95.96	98.81	1.00
	VNet	✓		✓	95.70	95.90	98.87	1.00
	VNet		✓	✓	95.72	95.92	98.88	0.00
	UNet++	✓			95.99	96.01	98.99	0.00
	UNet++		✓		96.97	97.03	99.22	0.00
	UNet++			✓	95.99	96.01	98.99	0.00
	UNet++	✓		✓	97.12	97.67	99.13	0.00
	UNet++		✓	✓	97.67	98.14	99.29	0.00
	ResUNet-a	✓			95.99	96.02	98.99	0.00
	ResUNet-a		✓		96.00	96.02	98.99	0.00
	ResUNet-a			✓	97.93	98.05	99.45	0.00
	ResUNet-a	✓		✓	96.05	96.07	99.01	0.00
	ResUNet-a		✓	✓	97.16	97.29	99.26	0.00
	U^2Net	✓			95.84	96.03	98.91	1.00
	U^2Net		✓		96.02	96.21	98.95	1.00
	U^2Net			✓	95.99	96.01	98.99	1.00
	U^2Net	✓		✓	96.06	93.79	99.63	0.00
	U^2Net		✓	✓	96.92	96.31	99.39	0.00
Fully Attention Baselines	Att UNet	✓			93.82	93.82	98.46	1.00
	Att UNet		✓		94.13	93.89	98.60	1.00
	Att UNet			✓	94.04	93.89	98.56	1.00
	Att UNet	✓		✓	94.15	94.06	98.56	1.00
	Att UNet		✓	✓	95.73	95.73	98.94	0.00
	TransUNet	✓			95.81	96.39	98.80	0.00
	TransUNet		✓		97.05	97.20	99.22	0.00
	TransUNet			✓	95.81	96.39	98.79	0.00
	TransUNet	✓		✓	93.82	93.82	98.46	1.00
	TransUNet		✓	✓	97.63	97.69	99.40	0.00
	Swin-UNet	✓			96.79	97.37	99.04	0.00
	Swin-UNet		✓		96.86	97.19	99.13	0.00
	Swin-UNet			✓	97.55	97.99	99.27	0.00
	Swin-UNet	✓		✓	97.83	97.83	99.32	0.00
	Swin-UNet		✓	✓	97.43	97.75	99.28	0.00
Proposed	customized UNet	✓			95.97	96.14	98.95	0.00
	customized UNet		✓		97.56	97.84	99.32	0.00
	customized UNet			✓	97.25	97.82	99.16	0.00
	customized UNet	✓		✓	98.07	98.38	99.43	0.00
	customized UNet		✓	✓	**98.63**	98.56	**99.68**	**0.00**

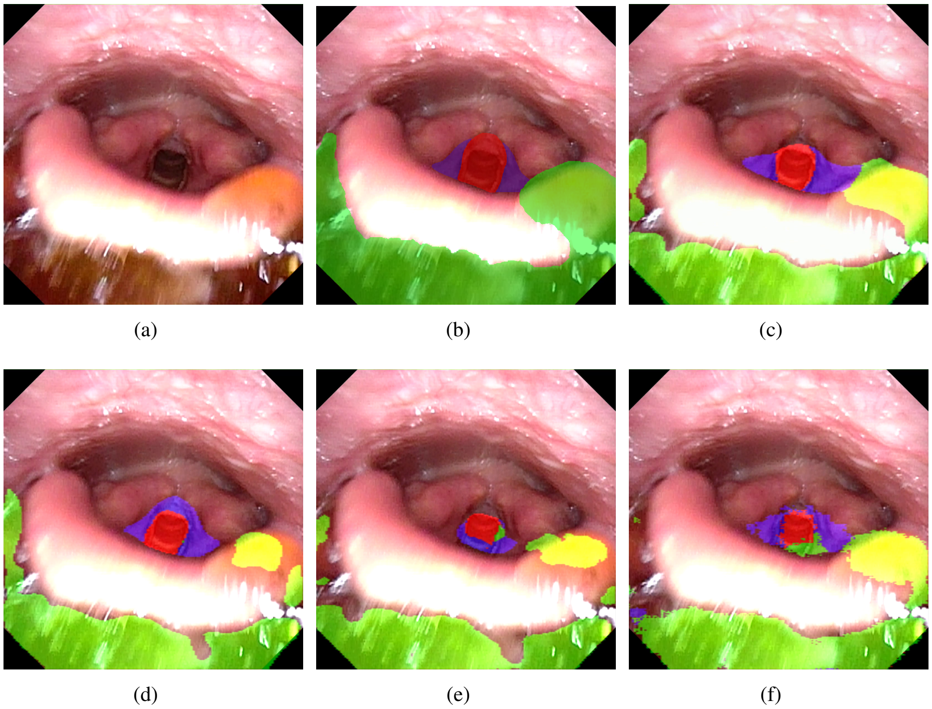

Figure S1. (a) A sample FEES image, its corresponding (b) ground truth, and a qualitative comparison of (c) customized UNet, (d) DeepLabv3+, (e) TransUNet, and (f) Swin-UNet. The proposed method achieves desirable segmentation results close to ground truth.

$$\mathscr{L}_{\text{CCE}}(y,p) = -\frac{1}{N}\sum_{c=1}^{C}\sum_{i=1}^{N} y_{ci}\cdot\log\left(p_{ci}\right). \quad (3)$$

To choose a suitable loss function, we performed evaluations under four scenarios: $\mathscr{L}_{\text{GD}}$, $\mathscr{L}_{\text{GT}}$, a combination of $\mathscr{L}_{\text{GD}}$ and $\mathscr{L}_{\text{CCE}}$ ($\mathscr{L}_{\text{CCE+GD}}$), and a combination of $\mathscr{L}_{\text{GT}}$ and $\mathscr{L}_{\text{CCE}}$ ($\mathscr{L}_{\text{CCE+GT}}$). All network minimize loss with the adaptive moment estimation (ADAM) optimizer[12]. All networks were trained for 100 epochs using an ADAM optimizer with a batch size of 8 because neither of the networks improved after 100 epochs. The initial learning rate is dependent on the networks and decays $1e$-4 if validation loss does not improve after four epochs.

RESULTS

The quantitative segmentation performance are evaluated by the $Sensitivity$ (%), $Specificity$ (%), $Dice\ similarity\ coefficient$ (DSC, %), and 95% $Hausdorff\ distance$(HD95) defined in the main paper.

Results on Test FEES Videos

The segmentation performance of the customized UNet and the comparison with other networks are shown in Table S1. We have four major observations from Table 1. First, the customized UNet is the best network for segmenting FEES videos in our experiments. The customized UNet trained with $\mathscr{L}_{\text{CCE+GT}}$ achieved superior performance to other netowrks in terms of DSC and specificity. Second, we observed that a loss function for a given network is important. In terms of DSC, HRNet and ResUNet-a achieved their best segmentation performance with $\mathscr{L}_{\text{CCE}}$, Swin-UNet achieved its best segmentation performance with $\mathscr{L}_{\text{CCE+GD}}$, DeepLabv3+, VNet, UNet++, U^2Net, Att UNet, TransUNet, and the customized UNet achieved their best segmentation performance with $\mathscr{L}_{\text{CCE+GT}}$, respectively. Third, balancing between the sensitivity and specificity is important. DeepLabv3+ trained with $\mathscr{L}_{\text{CCE+GT}}$ attained the highest sensitivity of 99.58 % and low specificity, resulting in lower DSC than the customized UNet. Fourth, modifying the original UNet for binary medical segmentation may turn out to reduce the segmentation performance on multiclass segmentation tasks. Convolutional VNet, UNet++, ResUNet-a, and U^2Net and fully attention baselines Att UNet, TransUNet, and Swin-UNet had less segmentation performance to varying degrees compared with

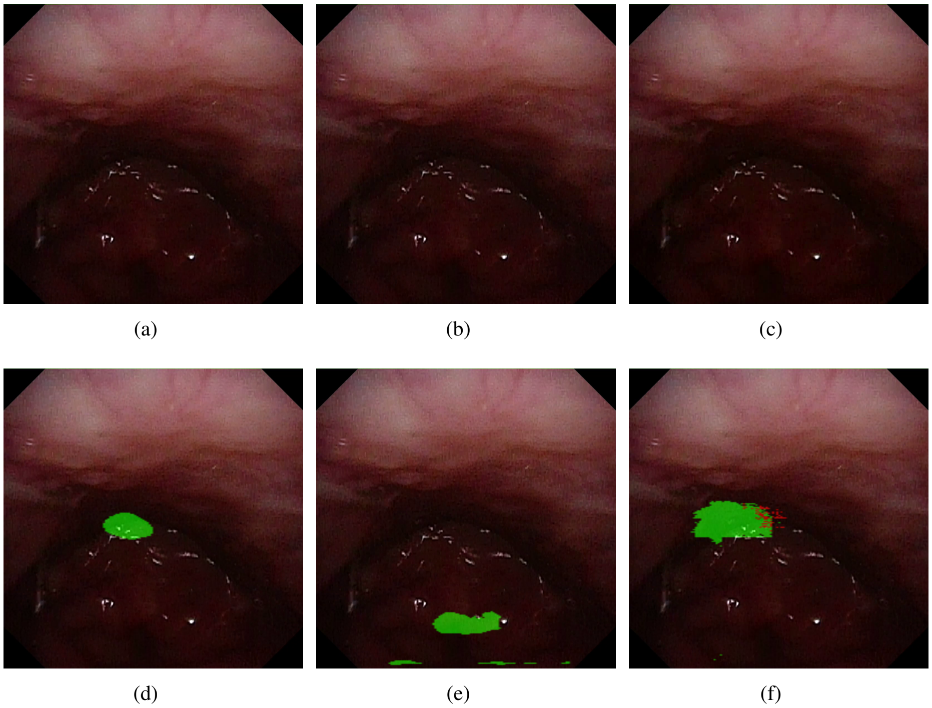

Figure S2. (*a*) A sample FEES image, corresponding (*b*) ground truth, and qualitative comparison between (*c*) customized UNet, (*d*) DeepLabv3+, (*e*) TransUNet, and (*f*) Swin-UNet. The proposed method achieves desirable segmentation results which are close to ground truth.

the orginal UNet. Overly complicated variants of UNet would lead to overfitting. A simple network should be given a higher preference in our multiclass segmentation task. The aforementioned UNet variants have improved segmentation performance over UNet via using ensemble of multiple blocks like Dense-like block, Inception-like block, or transformer block. ResUNet-a only includes residual connections to avoid overfitting. ResUNet-a achieved the highest DSC among the aforementioned UNet variants. This may indicate overfitting is a serious problem in FEES video segmentation.

Discussion

Fig. 1 shows an example of FEES image 1(a), its corresponding segmentation result 1(b), and segmentation results from customized UNet 1(c), DeepLabv3+ 1(d), TransUNet 1(e), and Swin-UNet 1(f). The image was extracted from a test video and the customized UNet outperformed all baselines. This test video was from a patient with no penetration, aspiration, or residue in the hypopharynx. The customized UNet successfully segmented the residue in the patient's vallecula, and also predicted no residue in the aspiration area or penetration area. Baseline networks, by contrast, predicted there were test bolus stuck in the aspiration area (Deeplabv3+, TransUNet, and Swin-UNet) or (TransUNet and Swin-UNet) penetration area. As shown in Table 1, Deeplabv3+ achieved the second-best DSC. In this example, it also reached the second-best segmentation result. Deeplabv3+ only wrongly segmented some pixels in the aspiration area as residue, which had little impact on judgment making by laryngologists. In contrast, TransUNet and Swin-UNet wrongly segmented a lot of pixels in the aspiration area and penetration area. Segmentation of test bolus increased the contrast of fluids in the video recording during FEES, and thus enhanced the detection of aspiration and penetration. While the instant green "flickering" inside the hypopharynx or vallecula could be ignored, the instant green "flickering" appeared in the aspiration area or penetration area could lead to very different judgment of aspiration and penetration. A network with a high specificity should be given a higher preference due to practical consideration.

The endoscope is an essential part of FEES. Illumination has always been a great challenge for every type of endoscope and is now more important than ever for a network because using images under proper illumination can make learning easier (e.g., avoid low contrast and use structured light to obtain depth information)[13]. However, it is inevitable that many images are captured under poor illumination conditions in a FEES image sequence. Fig. 2 shows an example of an improperly illuminated FEES image 2(a) that exhibited characteristics like low brightness and low contrast. In this example with no residue in the aspiration area, penetration area, hypopharynx, and vallecula, the customized UNet 2(c) achieved a desirable segmentation result

which is the same as the ground truth 2(b). On the contrary, DeepLabv3+ 2(d) and Swin-UNet 2(f) incorrectly classified some pixels in the hypopharynx as test bolus, and TransUNet 2(e) wrongly predicted some pixels in the aspiration and penetration areas as test bolus. Segmentation increases the contrast of the test bolus in the FEES image. The false prediction of test bolus in a low contrast image is particularly attractive and may lead to improper judgment. None class pixels occupied many frames in FEES videos. This again indicates that a network with a simple architecture and high specificity is desired for practical consideration.

Concurrent Works

HRNet and DeepLabv3+ can maintain high-resolution representations. VNet, UNet++, ResUNet-a, and U^2Net can learn rich multi-scale contextual information from the mixture of receptive fields of different sizes. Transformer-based UNet variants Att UNet, TransUNet, and Swin-UNet can capture both global and long-range semantic information interactions. It was observed that the aforementioned network is not feasible for the FEES videos segmentation which is a multiclass class-imbalance problem. Unlike these works, we confirmed the feasibility of simply applying an encoder-decoder with skip connections for FEES image segmentation without needing pre-training. Quantitative and qualitative results imply that the customized UNet is suitable for supporting diagnosis decision-making in FEES videos.

References

1. Wang, J. *et al.* Deep high-resolution representation learning for visual recognition. *IEEE transactions on pattern analysis machine intelligence* **43**, 3349–3364 (2020).
2. Chen, L.-C., Zhu, Y., Papandreou, G., Schroff, F. & Adam, H. Encoder-decoder with atrous separable convolution for semantic image segmentation. In *Proceedings of the European conference on computer vision (ECCV)*, 801–818 (2018).
3. Milletari, F., Navab, N. & Ahmadi, S.-A. V-net: Fully convolutional neural networks for volumetric medical image segmentation. In *2016 fourth international conference on 3D vision (3DV)*, 565–571 (IEEE, 2016).
4. Zhou, Z., Siddiquee, M. M. R., Tajbakhsh, N. & Liang, J. Unet++: Redesigning skip connections to exploit multiscale features in image segmentation. *IEEE transactions on medical imaging* **39**, 1856–1867 (2019).
5. Diakogiannis, F. I., Waldner, F., Caccetta, P. & Wu, C. Resunet-a: A deep learning framework for semantic segmentation of remotely sensed data. *ISPRS J. Photogramm. Remote. Sens.* **162**, 94–114 (2020).
6. Qin, X. *et al.* U2-net: Going deeper with nested u-structure for salient object detection. *Pattern recognition* **106**, 107404 (2020).
7. Oktay, O. *et al.* Attention u-net: Learning where to look for the pancreas. *arXiv preprint arXiv:1804.03999* (2018).
8. Chen, J. *et al.* Transunet: Transformers make strong encoders for medical image segmentation. *arXiv preprint arXiv:2102.04306* (2021).
9. Cao, H. *et al.* Swin-unet: Unet-like pure transformer for medical image segmentation. *arXiv preprint arXiv:2105.05537* (2021).
10. He, K., Zhang, X., Ren, S. & Sun, J. Delving deep into rectifiers: Surpassing human-level performance on imagenet classification. In *Proceedings of the IEEE international conference on computer vision*, 1026–1034 (2015).
11. Sudre, C. H., Li, W., Vercauteren, T., Ourselin, S. & Jorge Cardoso, M. Generalised dice overlap as a deep learning loss function for highly unbalanced segmentations. In *Deep learning in medical image analysis and multimodal learning for clinical decision support*, 240–248 (Springer, 2017).
12. Kingma, D. P. & Ba, J. Adam: A method for stochastic optimization. *arXiv preprint arXiv:1412.6980* (2014).
13. Dodge, S. & Karam, L. Understanding how image quality affects deep neural networks. In *2016 eighth international conference on quality of multimedia experience (QoMEX)*, 1–6 (IEEE, 2016).

Acknowledgements

This study was partially supported by the Competitive Research Fund, The University of Aizu (2022-P-12).

Author contributions statement

W.W., M.I., and S.M. carried out the experiments; W.W. wrote the manuscript with support from M.I. and X.Z.; W.W., M.I., and X.Z. conceived the original idea; W.W., M.I., S.M., and X.Z. analyzed the results; M.I. and X.Z. supervised the project. All authors reviewed the manuscript.

Additional information

Competing interests The authors declare no competing interests.
