# Supplementary material for: Expert-level aspiration and penetration detection during flexible endoscopic evaluation of swallowing with artificial intelligence-assisted diagnosis
Source: Sci Rep. 2022 Dec 15;12:21689. doi: 10.1038/s41598-022-25618-z (PMC9753025; doi:10.1038/s41598-022-25618-z)
Supplement: Supplementary file 3 — Supplementary Legends. [file 41598_2022_25618_MOESM3_ESM.docx]

Video S1. A video clip from an original video recorded during the flexible endoscopic evaluation of swallowing.
